# Where the International Brazilian Journal of Urology is going in 2026

**DOI:** 10.1590/S1677-5538.IBJU.2026.01.01

**Published:** 2026-02-02

**Authors:** Luciano A. Favorito

**Affiliations:** 1 Universidade do Estado do Rio de Janeiro Unidade de Pesquisa Urogenital Rio de Janeiro RJ Brasil Unidade de Pesquisa Urogenital - Universidade do Estado do Rio de Janeiro - Uerj, Rio de Janeiro, RJ, Brasil; 2 Hospital Federal da Lagoa Serviço de Urologia Rio de Janeiro RJ Brasil Serviço de Urologia, Hospital Federal da Lagoa, Rio de Janeiro, RJ, Brasil

The January–February 2026 issue of the International Brazilian Journal of Urology marks the beginning of a very important year. In the most recent evaluation, we achieved the highest impact factor in our history (4.5), consolidating our position among the ten most influential urology journals in the world.

For 2026, our challenge is to further increase the journal's visibility and scientific relevance. To achieve this, we will need to work even harder and continue to strengthen the rigor of our editorial and peer review processes. Our goal is to raise the impact factor even higher and bring the International Brazilian Journal of Urology to the very top tier of urological publications worldwide.

This issue presents several original and noteworthy contributions across different fields of urology, with special emphasis on reconstructive urology — the focus of our cover article (1). Dr. Huang and colleagues from China present an interesting study (page e20250385) evaluating the efficacy and safety of robotic-assisted bladder neck reconstruction in patients with refractory vesicourethral anastomotic stenosis (VUAS) following radical prostatectomy (RP) or radical cystectomy (RC) with orthotopic neobladder (ONB) reconstruction. The authors conclude that robotic-assisted bladder neck reconstruction is a safe and effective surgical option, achieving high patency rates and low incidence of de novo stress urinary incontinence in this challenging patient population.

Over the past two years, the International Brazilian Journal of Urology has published several important studies in reconstructive urology (2-5), reflecting the growing importance of this field in contemporary urological practice.

The Editor-in-Chief hopes you enjoy reading this issue.
